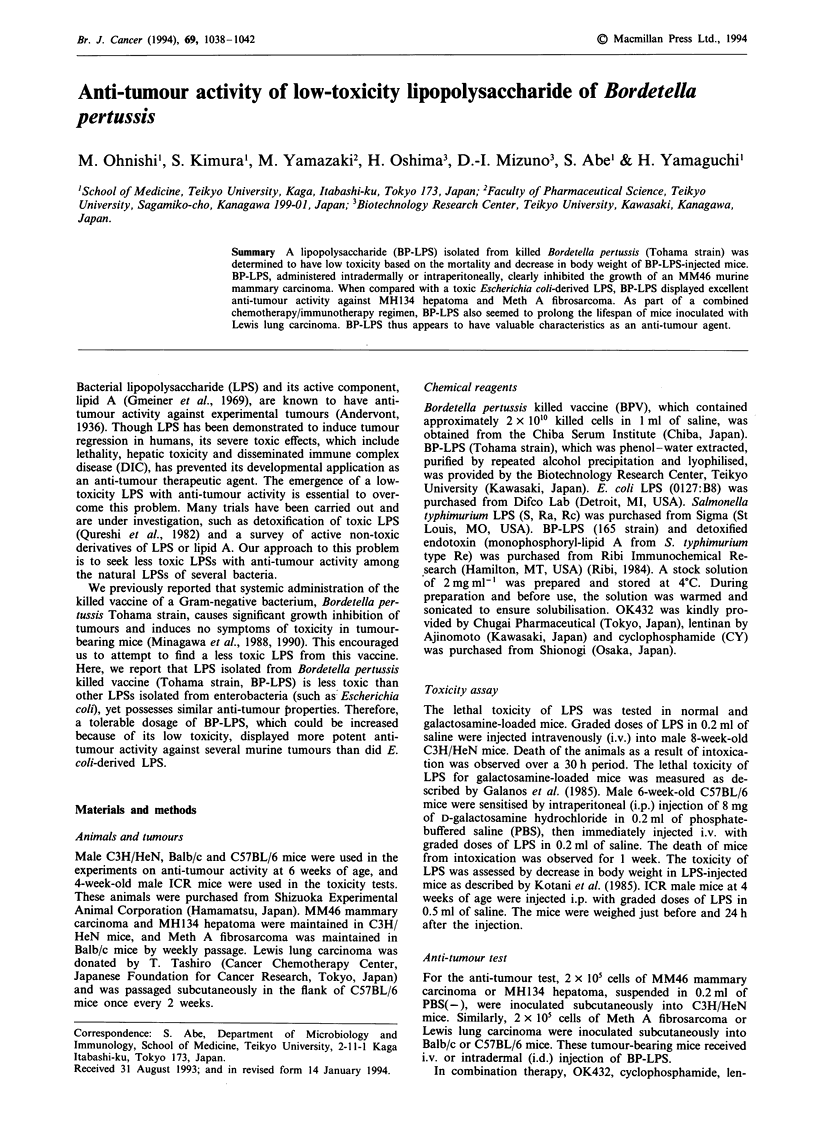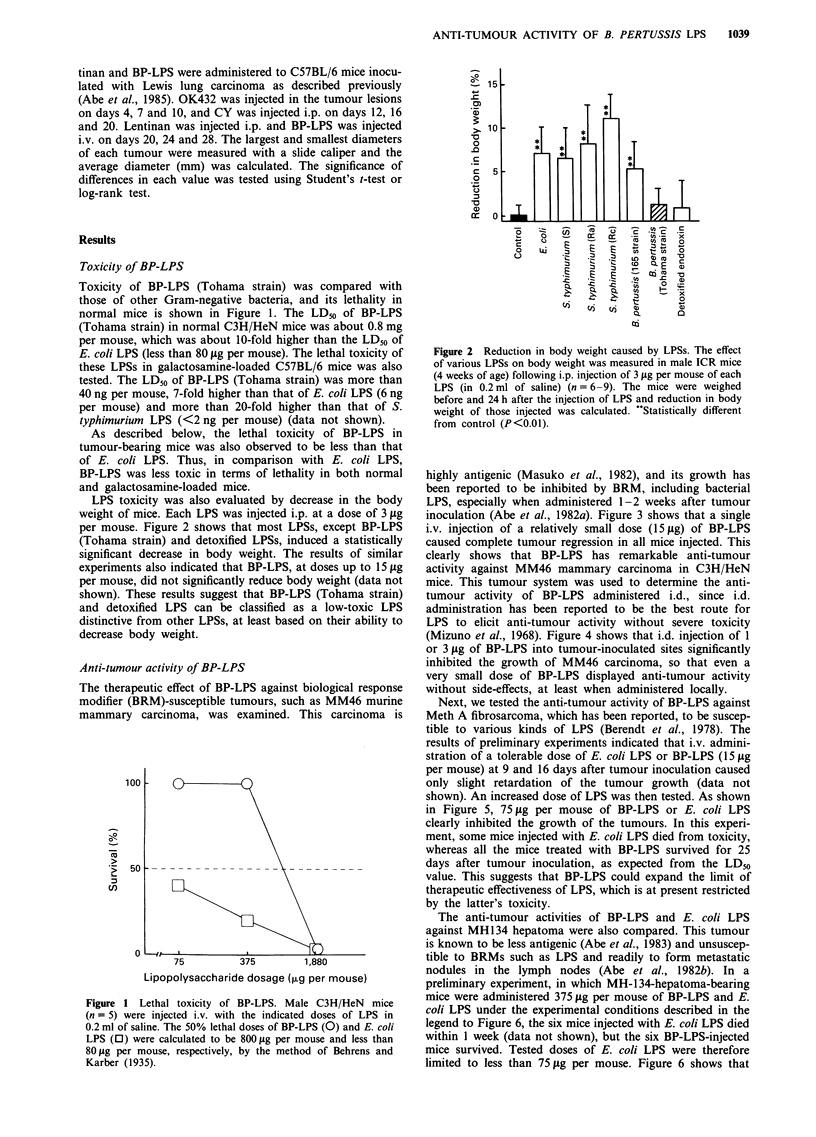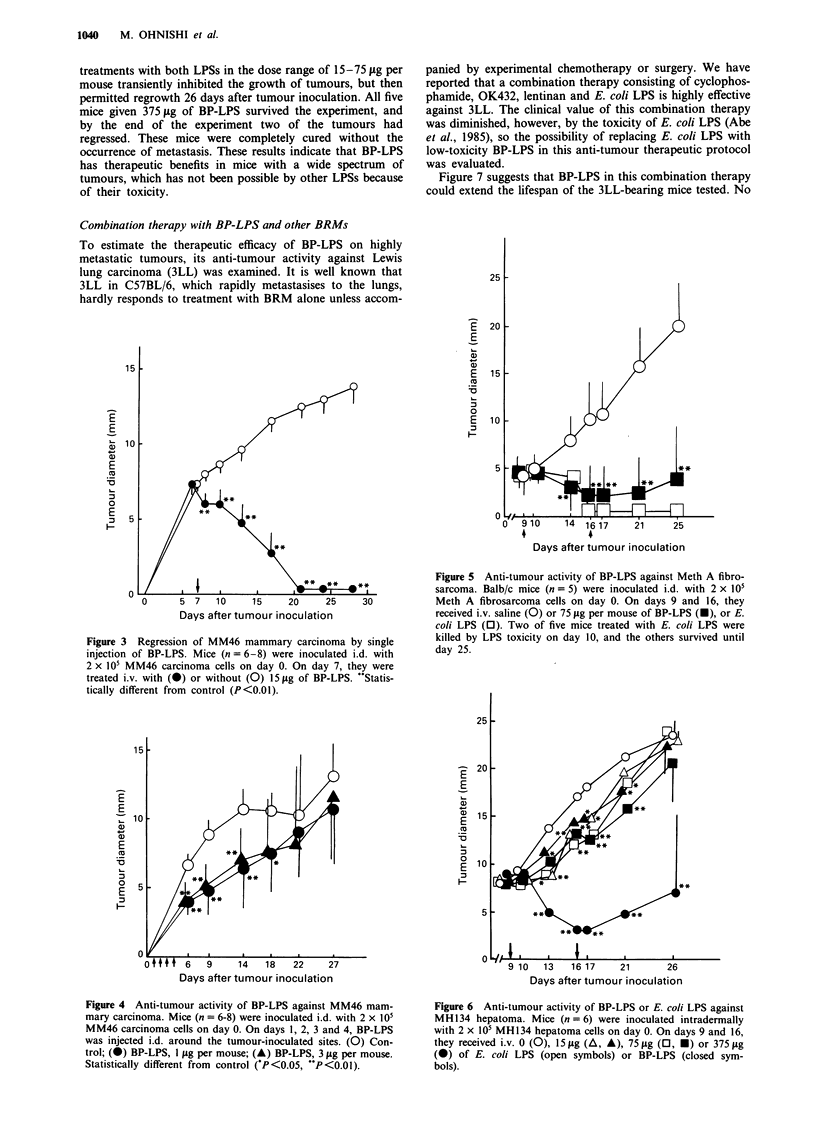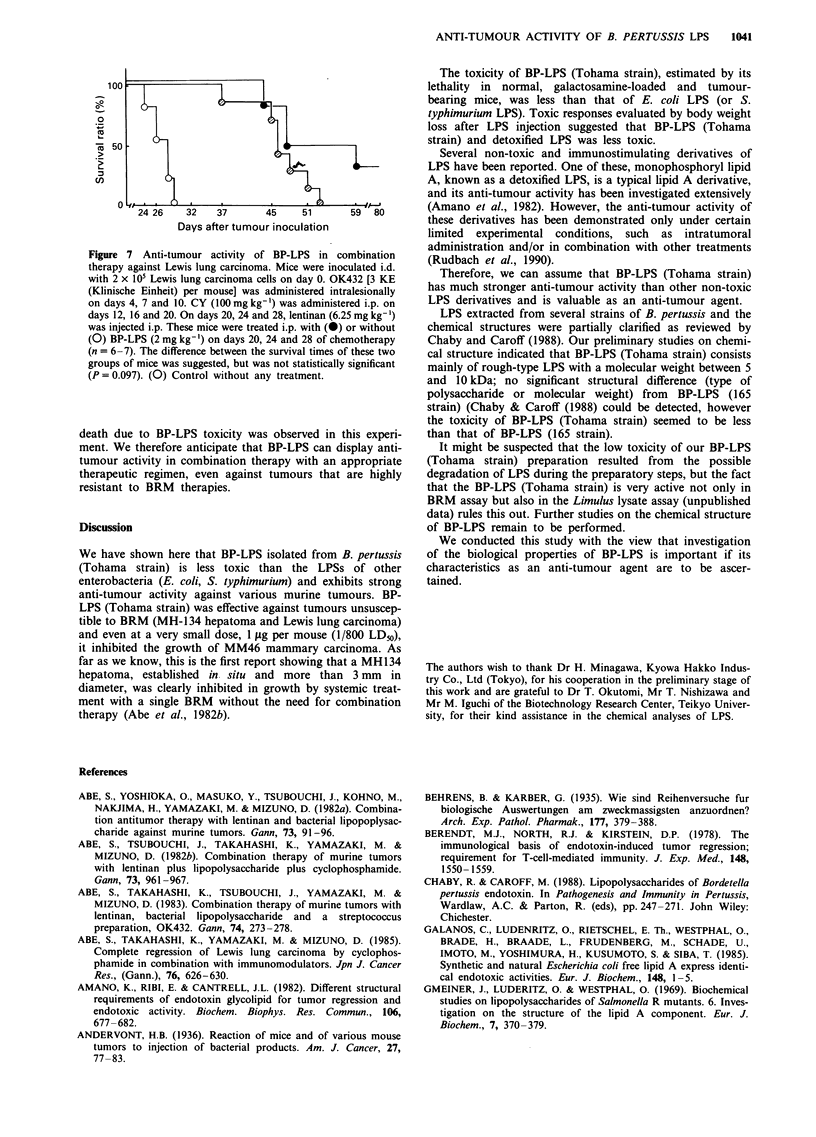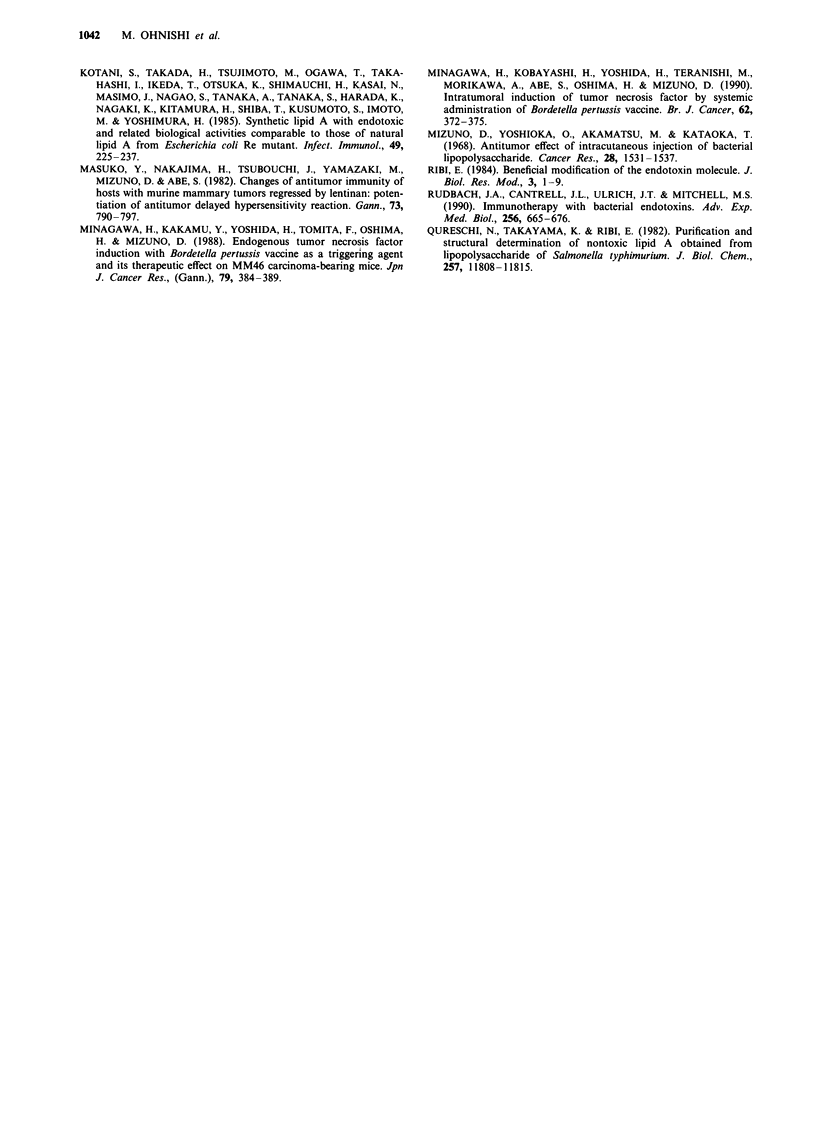# Anti-tumour activity of low-toxicity lipopolysaccharide of Bordetella pertussis.

**DOI:** 10.1038/bjc.1994.204

**Published:** 1994-06

**Authors:** M. Ohnishi, S. Kimura, M. Yamazaki, H. Oshima, D. I. Mizuno, S. Abe, H. Yamaguchi

**Affiliations:** School of Medicine, Teikyo University, Tokyo, Japan.

## Abstract

A lipopolysaccharide (BP-LPS) isolated from killed Bordetella pertussis (Tohama strain) was determined to have low toxicity based on the mortality and decrease in body weight of BP-LPS-injected mice. BP-LPS, administered intradermally or intraperitoneally, clearly inhibited the growth of an MM46 murine mammary carcinoma. When compared with a toxic Escherichia coli-derived LPS, BP-LPS displayed excellent anti-tumour activity against MH134 hepatoma and Meth A fibrosarcoma. As part of a combined chemotherapy/immunotherapy regimen, BP-LPS also seemed to prolong the lifespan of mice inoculated with Lewis lung carcinoma. BP-LPS thus appears to have valuable characteristics as an anti-tumour agent.


 © Macmillan Press Ltd., 1994

### Anti-tumour activity of low-toxicity lipopolysaccharide of *Bordetella pertussis*

M. Ohnishi[1], S. Kimura[1], M. Yamazaki[2], H. Oshima[3], D.-I. Mizuno[3], S. Abe[1] & H. Yamaguchi[1]

[1]*School of Medicine, Teikyo University, Kaga, Itabashi-ku, Tokyo 173, Japan;* [2]*Faculty of Pharmaceutical Science, Teikyo University, Sagamiko-cho, Kanagawa 199-01, Japan;* [3]*Biotechnology Research Center, Teikyo University, Kawasaki, Kanagawa, Japan.*

**Summary** A lipopolysaccharide (BP-LPS) isolated from killed *Bordetella pertussis* (Tohama strain) was determined to have low toxicity based on the mortality and decrease in body weight of BP-LPS-injected mice. BP-LPS, administered intradermally or intraperitoneally, clearly inhibited the growth of an MM46 murine mammary carcinoma. When compared with a toxic *Escherichia coli*-derived LPS, BP-LPS displayed excellent anti-tumour activity against MH134 hepatoma and Meth A fibrosarcoma. As part of a combined chemotherapy/immunotherapy regimen, BP-LPS also seemed to prolong the lifespan of mice inoculated with Lewis lung carcinoma. BP-LPS thus appears to have valuable characteristics as an anti-tumour agent.

Bacterial lipopolysaccharide (LPS) and its active component, lipid A (Gmeiner *et al.*, 1969), are known to have anti-tumour activity against experimental tumours (Andervont, 1936). Though LPS has been demonstrated to induce tumour regression in humans, its severe toxic effects, which include lethality, hepatic toxicity and disseminated immune complex disease (DIC), has prevented its developmental application as an anti-tumour therapeutic agent. The emergence of a low-toxicity LPS with anti-tumour activity is essential to overcome this problem. Many trials have been carried out and are under investigation, such as detoxification of toxic LPS (Qureshi *et al.*, 1982) and a survey of active non-toxic derivatives of LPS or lipid A. Our approach to this problem is to seek less toxic LPSs with anti-tumour activity among the natural LPSs of several bacteria.

We previously reported that systemic administration of the killed vaccine of a Gram-negative bacterium, *Bordetella pertussis* Tohama strain, causes significant growth inhibition of tumours and induces no symptoms of toxicity in tumour-bearing mice (Minagawa *et al.*, 1988, 1990). This encouraged us to attempt to find a less toxic LPS from this vaccine. Here, we report that LPS isolated from *Bordetella pertussis* killed vaccine (Tohama strain, BP-LPS) is less toxic than other LPSs isolated from enterobacteria (such as *Escherichia coli*), yet possesses similar anti-tumour properties. Therefore, a tolerable dosage of BP-LPS, which could be increased because of its low toxicity, displayed more potent anti-tumour activity against several murine tumours than did *E. coli*-derived LPS.

### Materials and methods

### *Animals and tumours*

Male C3H/HeN, Balb/c and C57BL/6 mice were used in the experiments on anti-tumour activity at 6 weeks of age, and 4-week-old male ICR mice were used in the toxicity tests. These animals were purchased from Shizuoka Experimental Animal Corporation (Hamamatsu, Japan). MM46 mammary carcinoma and MH134 hepatoma were maintained in C3H/HeN mice, and Meth A fibrosarcoma was maintained in Balb/c mice by weekly passage. Lewis lung carcinoma was donated by T. Tashiro (Cancer Chemotherapy Center, Japanese Foundation for Cancer Research, Tokyo, Japan) and was passaged subcutaneously in the flank of C57BL/6 mice once every 2 weeks.

Correspondence: S. Abe, Department of Microbiology and Immunology, School of Medicine, Teikyo University, 2-11-1 Kaga Itabashi-ku, Tokyo 173, Japan.
Received 31 August 1993; and in revised form 14 January 1994.

### *Chemical reagents*

*Bordetella pertussis* killed vaccine (BPV), which contained approximately $2 \times 10^{10}$ killed cells in 1 ml of saline, was obtained from the Chiba Serum Institute (Chiba, Japan). BP-LPS (Tohama strain), which was phenol–water extracted, purified by repeated alcohol precipitation and lyophilised, was provided by the Biotechnology Research Center, Teikyo University (Kawasaki, Japan). *E. coli* LPS (0127:B8) was purchased from Difco Lab (Detroit, MI, USA). *Salmonella typhimurium* LPS (S, Ra, Rc) was purchased from Sigma (St Louis, MO, USA). BP-LPS (165 strain) and detoxified endotoxin (monophosphoryl-lipid A from *S. typhimurium* type Re) was purchased from Ribi Immunochemical Research (Hamilton, MT, USA) (Ribi, 1984). A stock solution of 2 mg $ml^{-1}$ was prepared and stored at 4°C. During preparation and before use, the solution was warmed and sonicated to ensure solubilisation. OK432 was kindly provided by Chugai Pharmaceutical (Tokyo, Japan), lentinan by Ajinomoto (Kawasaki, Japan) and cyclophosphamide (CY) was purchased from Shionogi (Osaka, Japan).

### *Toxicity assay*

The lethal toxicity of LPS was tested in normal and galactosamine-loaded mice. Graded doses of LPS in 0.2 ml of saline were injected intravenously (i.v.) into male 8-week-old C3H/HeN mice. Death of the animals as a result of intoxication was observed over a 30 h period. The lethal toxicity of LPS for galactosamine-loaded mice was measured as described by Galanos *et al.* (1985). Male 6-week-old C57BL/6 mice were sensitised by intraperitoneal (i.p.) injection of 8 mg of D-galactosamine hydrochloride in 0.2 ml of phosphate-buffered saline (PBS), then immediately injected i.v. with graded doses of LPS in 0.2 ml of saline. The death of mice from intoxication was observed for 1 week. The toxicity of LPS was assessed by decrease in body weight in LPS-injected mice as described by Kotani *et al.* (1985). ICR male mice at 4 weeks of age were injected i.p. with graded doses of LPS in 0.5 ml of saline. The mice were weighed just before and 24 h after the injection.

### *Anti-tumour test*

For the anti-tumour test, $2 \times 10^5$ cells of MM46 mammary carcinoma or MH134 hepatoma, suspended in 0.2 ml of PBS(−), were inoculated subcutaneously into C3H/HeN mice. Similarly, $2 \times 10^5$ cells of Meth A fibrosarcoma or Lewis lung carcinoma were inoculated subcutaneously into Balb/c or C57BL/6 mice. These tumour-bearing mice received i.v. or intradermal (i.d.) injection of BP-LPS.

In combination therapy, OK432, cyclophosphamide, lentinan and BP-LPS were administered to C57BL/6 mice inoculated with Lewis lung carcinoma as described previously (Abe *et al.*, 1985). OK432 was injected in the tumour lesions on days 4, 7 and 10, and CY was injected i.p. on days 12, 16 and 20. Lentinan was injected i.p. and BP-LPS was injected i.v. on days 20, 24 and 28. The largest and smallest diameters of each tumour were measured with a slide caliper and the average diameter (mm) was calculated. The significance of differences in each value was tested using Student's *t*-test or log-rank test.

### Results

### *Toxicity of BP-LPS*

Toxicity of BP-LPS (Tohama strain) was compared with those of other Gram-negative bacteria, and its lethality in normal mice is shown in Figure 1. The $LD_{50}$ of BP-LPS (Tohama strain) in normal C3H/HeN mice was about 0.8 mg per mouse, which was about 10-fold higher than the $LD_{50}$ of *E. coli* LPS (less than 80 μg per mouse). The lethal toxicity of these LPSs in galactosamine-loaded C57BL/6 mice was also tested. The $LD_{50}$ of BP-LPS (Tohama strain) was more than 40 ng per mouse, 7-fold higher than that of *E. coli* LPS (6 ng per mouse) and more than 20-fold higher than that of *S. typhimurium* LPS (<2 ng per mouse) (data not shown).

As described below, the lethal toxicity of BP-LPS in tumour-bearing mice was also observed to be less than that of *E. coli* LPS. Thus, in comparison with *E. coli* LPS, BP-LPS was less toxic in terms of lethality in both normal and galactosamine-loaded mice.

LPS toxicity was also evaluated by decrease in the body weight of mice. Each LPS was injected i.p. at a dose of 3 μg per mouse. Figure 2 shows that most LPSs, except BP-LPS (Tohama strain) and detoxified LPSs, induced a statistically significant decrease in body weight. The results of similar experiments also indicated that BP-LPS, at doses up to 15 μg per mouse, did not significantly reduce body weight (data not shown). These results suggest that BP-LPS (Tohama strain) and detoxified LPS can be classified as a low-toxic LPS distinctive from other LPSs, at least based on their ability to decrease body weight.

### *Anti-tumour activity of BP-LPS*

The therapeutic effect of BP-LPS against biological response modifier (BRM)-susceptible tumours, such as MM46 murine mammary carcinoma, was examined. This carcinoma is

**Figure 1** Lethal toxicity of BP-LPS. Male C3H/HeN mice ($n = 5$) were injected i.v. with the indicated doses of LPS in 0.2 ml of saline. The 50% lethal doses of BP-LPS (○) and *E. coli* LPS (□) were calculated to be 800 μg per mouse and less than 80 μg per mouse, respectively, by the method of Behrens and Karber (1935).

**Figure 2** Reduction in body weight caused by LPSs. The effect of various LPSs on body weight was measured in male ICR mice (4 weeks of age) following i.p. injection of 3 μg per mouse of each LPS (in 0.2 ml of saline) ($n = 6-9$). The mice were weighed before and 24 h after the injection of LPS and reduction in body weight of those injected was calculated. **Statistically different from control ($P < 0.01$).

highly antigenic (Masuko *et al.*, 1982), and its growth has been reported to be inhibited by BRM, including bacterial LPS, especially when administered 1–2 weeks after tumour inoculation (Abe *et al.*, 1982*a*). Figure 3 shows that a single i.v. injection of a relatively small dose (15 μg) of BP-LPS caused complete tumour regression in all mice injected. This clearly shows that BP-LPS has remarkable anti-tumour activity against MM46 mammary carcinoma in C3H/HeN mice. This tumour system was used to determine the anti-tumour activity of BP-LPS administered i.d., since i.d. administration has been reported to be the best route for LPS to elicit anti-tumour activity without severe toxicity (Mizuno *et al.*, 1968). Figure 4 shows that i.d. injection of 1 or 3 μg of BP-LPS into tumour-inoculated sites significantly inhibited the growth of MM46 carcinoma, so that even a very small dose of BP-LPS displayed anti-tumour activity without side-effects, at least when administered locally.

Next, we tested the anti-tumour activity of BP-LPS against Meth A fibrosarcoma, which has been reported, to be susceptible to various kinds of LPS (Berendt *et al.*, 1978). The results of preliminary experiments indicated that i.v. administration of a tolerable dose of *E. coli* LPS or BP-LPS (15 μg per mouse) at 9 and 16 days after tumour inoculation caused only slight retardation of the tumour growth (data not shown). An increased dose of LPS was then tested. As shown in Figure 5, 75 μg per mouse of BP-LPS or *E. coli* LPS clearly inhibited the growth of the tumours. In this experiment, some mice injected with *E. coli* LPS died from toxicity, whereas all the mice treated with BP-LPS survived for 25 days after tumour inoculation, as expected from the $LD_{50}$ value. This suggests that BP-LPS could expand the limit of therapeutic effectiveness of LPS, which is at present restricted by the latter's toxicity.

The anti-tumour activities of BP-LPS and *E. coli* LPS against MH134 hepatoma were also compared. This tumour is known to be less antigenic (Abe *et al.*, 1983) and unsusceptible to BRMs such as LPS and readily to form metastatic nodules in the lymph nodes (Abe *et al.*, 1982*b*). In a preliminary experiment, in which MH-134-hepatoma-bearing mice were administered 375 μg per mouse of BP-LPS and *E. coli* LPS under the experimental conditions described in the legend to Figure 6, the six mice injected with *E. coli* LPS died within 1 week (data not shown), but the six BP-LPS-injected mice survived. Tested doses of *E. coli* LPS were therefore limited to less than 75 μg per mouse. Figure 6 shows that treatments with both LPSs in the dose range of 15–75 µg per mouse transiently inhibited the growth of tumours, but then permitted regrowth 26 days after tumour inoculation. All five mice given 375 µg of BP-LPS survived the experiment, and by the end of the experiment two of the tumours had regressed. These mice were completely cured without the occurrence of metastasis. These results indicate that BP-LPS has therapeutic benefits in mice with a wide spectrum of tumours, which has not been possible by other LPSs because of their toxicity.

### *Combination therapy with BP-LPS and other BRMs*

To estimate the therapeutic efficacy of BP-LPS on highly metastatic tumours, its anti-tumour activity against Lewis lung carcinoma (3LL) was examined. It is well known that 3LL in C57BL/6, which rapidly metastasises to the lungs, hardly responds to treatment with BRM alone unless accompanied by experimental chemotherapy or surgery. We have reported that a combination therapy consisting of cyclophosphamide, OK432, lentinan and *E. coli* LPS is highly effective against 3LL. The clinical value of this combination therapy was diminished, however, by the toxicity of *E. coli* LPS (Abe *et al.*, 1985), so the possibility of replacing *E. coli* LPS with low-toxicity BP-LPS in this anti-tumour therapeutic protocol was evaluated.

Figure 7 suggests that BP-LPS in this combination therapy could extend the lifespan of the 3LL-bearing mice tested. No

**Figure 3** Regression of MM46 mammary carcinoma by single injection of BP-LPS. Mice ($n = 6–8$) were inoculated i.d. with $2 \times 10^5$ MM46 carcinoma cells on day 0. On day 7, they were treated i.v. with (●) or without (○) 15 µg of BP-LPS. **Statistically different from control ($P<0.01$).

**Figure 4** Anti-tumour activity of BP-LPS against MM46 mammary carcinoma. Mice ($n$ = 6-8) were inoculated i.d. with $2 \times 10^5$ MM46 carcinoma cells on day 0. On days 1, 2, 3 and 4, BP-LPS was injected i.d. around the tumour-inoculated sites. (○) Control; (●) BP-LPS, 1 µg per mouse; (▲) BP-LPS, 3 µg per mouse. Statistically different from control (*$P<0.05$, **$P<0.01$).

**Figure 5** Anti-tumour activity of BP-LPS against Meth A fibrosarcoma. Balb/c mice ($n = 5$) were inoculated i.d. with $2 \times 10^5$ Meth A fibrosarcoma cells on day 0. On days 9 and 16, they received i.v. saline (○) or 75 µg per mouse of BP-LPS (■), or *E. coli* LPS (□). Two of five mice treated with *E. coli* LPS were killed by LPS toxicity on day 10, and the others survived until day 25.

**Figure 6** Anti-tumour activity of BP-LPS or *E. coli* LPS against MH134 hepatoma. Mice ($n = 6$) were inoculated intradermally with $2 \times 10^5$ MH134 hepatoma cells on day 0. On days 9 and 16, they received i.v. 0 (○), 15 µg (△, ▲), 75 µg (□, ■) or 375 µg (●) of *E. coli* LPS (open symbols) or BP-LPS (closed symbols).

**Figure 7** Anti-tumour activity of BP-LPS in combination therapy against Lewis lung carcinoma. Mice were inoculated i.d. with $2 \times 10^5$ Lewis lung carcinoma cells on day 0. OK432 [3 KE (Klinische Einheit) per mouse] was administered intralesionally on days 4, 7 and 10. CY ($100 \text{ mg kg}^{-1}$) was administered i.p. on days 12, 16 and 20. On days 20, 24 and 28, lentinan ($6.25 \text{ mg kg}^{-1}$) was injected i.p. These mice were treated i.p. with (●) or without (○) BP-LPS ($2 \text{ mg kg}^{-1}$) on days 20, 24 and 28 of chemotherapy ($n = 6-7$). The difference between the survival times of these two groups of mice was suggested, but was not statistically significant ($P = 0.097$). (○) Control without any treatment.

death due to BP-LPS toxicity was observed in this experiment. We therefore anticipate that BP-LPS can display anti-tumour activity in combination therapy with an appropriate therapeutic regimen, even against tumours that are highly resistant to BRM therapies.

### Discussion

We have shown here that BP-LPS isolated from *B. pertussis* (Tohama strain) is less toxic than the LPSs of other enterobacteria (*E. coli*, *S. typhimurium*) and exhibits strong anti-tumour activity against various murine tumours. BP-LPS (Tohama strain) was effective against tumours unsusceptible to BRM (MH-134 hepatoma and Lewis lung carcinoma) and even at a very small dose, 1 μg per mouse (1/800 $LD_{50}$), it inhibited the growth of MM46 mammary carcinoma. As far as we know, this is the first report showing that a MH134 hepatoma, established *in situ* and more than 3 mm in diameter, was clearly inhibited in growth by systemic treatment with a single BRM without the need for combination therapy (Abe *et al.*, 1982*b*).

The toxicity of BP-LPS (Tohama strain), estimated by its lethality in normal, galactosamine-loaded and tumour-bearing mice, was less than that of *E. coli* LPS (or *S. typhimurium* LPS). Toxic responses evaluated by body weight loss after LPS injection suggested that BP-LPS (Tohama strain) and detoxified LPS was less toxic.

Several non-toxic and immunostimulating derivatives of LPS have been reported. One of these, monophosphoryl lipid A, known as a detoxified LPS, is a typical lipid A derivative, and its anti-tumour activity has been investigated extensively (Amano *et al.*, 1982). However, the anti-tumour activity of these derivatives has been demonstrated only under certain limited experimental conditions, such as intratumoral administration and/or in combination with other treatments (Rudbach *et al.*, 1990).

Therefore, we can assume that BP-LPS (Tohama strain) has much stronger anti-tumour activity than other non-toxic LPS derivatives and is valuable as an anti-tumour agent.

LPS extracted from several strains of *B. pertussis* and the chemical structures were partially clarified as reviewed by Chaby and Caroff (1988). Our preliminary studies on chemical structure indicated that BP-LPS (Tohama strain) consists mainly of rough-type LPS with a molecular weight between 5 and 10 kDa; no significant structural difference (type of polysaccharide or molecular weight) from BP-LPS (165 strain) (Chaby & Caroff (1988) could be detected, however the toxicity of BP-LPS (Tohama strain) seemed to be less than that of BP-LPS (165 strain).

It might be suspected that the low toxicity of our BP-LPS (Tohama strain) preparation resulted from the possible degradation of LPS during the preparatory steps, but the fact that the BP-LPS (Tohama strain) is very active not only in BRM assay but also in the *Limulus* lysate assay (unpublished data) rules this out. Further studies on the chemical structure of BP-LPS remain to be performed.

We conducted this study with the view that investigation of the biological properties of BP-LPS is important if its characteristics as an anti-tumour agent are to be ascertained.

The authors wish to thank Dr H. Minagawa, Kyowa Hakko Industry Co., Ltd (Tokyo), for his cooperation in the preliminary stage of this work and are grateful to Dr T. Okutomi, Mr T. Nishizawa and Mr M. Iguchi of the Biotechnology Research Center, Teikyo University, for their kind assistance in the chemical analyses of LPS.

### References

ABE, S., YOSHIOKA, O., MASUKO, Y., TSUBOUCHI, J., KOHNO, M., NAKJIMA, H., YAMAZAKI, M. & MIZUNO, D. (1982*a*). Combination antitumor therapy with lentinan and bacterial lipopoplysaccharide against murine tumors. *Gann*, **73**, 91-96.

ABE, S., TSUBOUCHI, J., TAKAHASHI, K., YAMAZAKI, M. & MIZUNO, D. (1982*b*). Combination therapy of murine tumors with lentinan plus lipopolysaccharide plus cyclophosphamide. *Gann*, **73**, 961-967.

ABE, S., TAKAHASHI, K., TSUBOUCHI, J., YAMAZAKI, M. & MIZUNO, D. (1983). Combination therapy of murine tumors with lentinan, bacterial lipopolysaccharide and a streptococcus preparation, OK432. *Gann*, **74**, 273-278.

ABE, S., TAKAHASHI, K., YAMAZAKI, M. & MIZUNO, D. (1985). Complete regression of Lewis lung carcinoma by cyclophosphamide in combination with immunomodulators. *Jpn J. Cancer Res.*, (Gann.), **76**, 626-630.

AMANO, K., RIBI, E. & CANTRELL, J.L. (1982). Different structural requirements of endotoxin glycolipid for tumor regression and endotoxic activity. *Biochem. Biophys. Res. Commun.*, **106**, 677-682.

ANDERVONT, H.B. (1936). Reaction of mice and of various mouse tumors to injection of bacterial products. *Am. J. Cancer*, **27**, 77-83.

BEHRENS, B. & KARBER, G. (1935). Wie sind Reihenversuche fur biologische Auswertungen am zweckmassigsten anzuordnen? *Arch. Exp. Pathol. Pharmak.*, **177**, 379-388.

BERENDT, M.J., NORTH, R.J. & KIRSTEIN, D.P. (1978). The immunological basis of endotoxin-induced tumor regression; requirement for T-cell-mediated immunity. *J. Exp. Med.*, **148**, 1550-1559.

CHABY, R. & CAROFF, M. (1988). Lipopolysaccharides of *Bordetella pertussis* endotoxin. In *Pathogenesis and Immunity in Pertussis*, Wardlaw, A.C. & Parton, R. (eds), pp. 247-271. John Wiley: Chichester.

GALANOS, C., LUDENRITZ, O., RIETSCHEL, E. Th., WESTPHAL, O., BRADE, H., BRAADE, L., FRUDENBERG, M., SCHADE, U., IMOTO, M., YOSHIMURA, H., KUSUMOTO, S. & SIBA, T. (1985). Synthetic and natural *Escherichia coli* free lipid A express identical endotoxic activities. *Eur. J. Biochem.*, **148**, 1-5.

GMEINER, J., LUDERITZ, O. & WESTPHAL, O. (1969). Biochemical studies on lipopolysaccharides of *Salmonella* R mutants. 6. Investigation on the structure of the lipid A component. *Eur. J. Biochem.*, **7**, 370-379.

KOTANI, S., TAKADA, H., TSUJIMOTO, M., OGAWA, T., TAKAHASHI, I., IKEDA, T., OTSUKA, K., SHIMAUCHI, H., KASAI, N., MASIMO, J., NAGAO, S., TANAKA, A., TANAKA, S., HARADA, K., NAGAKI, K., KITAMURA, H., SHIBA, T., KUSUMOTO, S., IMOTO, M. & YOSHIMURA, H. (1985). Synthetic lipid A with endotoxic and related biological activities comparable to those of natural lipid A from *Escherichia coli* Re mutant. *Infect. Immunol.*, **49**, 225–237.

MASUKO, Y., NAKAJIMA, H., TSUBOUCHI, J., YAMAZAKI, M., MIZUNO, D. & ABE, S. (1982). Changes of antitumor immunity of hosts with murine mammary tumors regressed by lentinan: potentiation of antitumor delayed hypersensitivity reaction. *Gann.*, **73**, 790–797.

MINAGAWA, H., KAKAMU, Y., YOSHIDA, H., TOMITA, F., OSHIMA, H. & MIZUNO, D. (1988). Endogenous tumor necrosis factor induction with *Bordetella pertussis* vaccine as a triggering agent and its therapeutic effect on MM46 carcinoma-bearing mice. *Jpn J. Cancer Res.*, (Gann.), **79**, 384–389.

MINAGAWA, H., KOBAYASHI, H., YOSHIDA, H., TERANISHI, M., MORIKAWA, A., ABE, S., OSHIMA, H. & MIZUNO, D. (1990). Intratumoral induction of tumor necrosis factor by systemic administration of *Bordetella pertussis* vaccine. *Br. J. Cancer*, **62**, 372–375.

MIZUNO, D., YOSHIOKA, O., AKAMATSU, M. & KATAOKA, T. (1968). Antitumor effect of intracutaneous injection of bacterial lipopolysaccharide. *Cancer Res.*, **28**, 1531–1537.

RIBI, E. (1984). Beneficial modification of the endotoxin molecule. *J. Biol. Res. Mod.*, **3**, 1–9.

RUDBACH, J.A., CANTRELL, J.L., ULRICH, J.T. & MITCHELL, M.S. (1990). Immunotherapy with bacterial endotoxins. *Adv. Exp. Med. Biol.*, **256**, 665–676.

QURESCHI, N., TAKAYAMA, K. & RIBI, E. (1982). Purification and structural determination of nontoxic lipid A obtained from lipopolysaccharide of *Salmonella typhimurium*. *J. Biol. Chem.*, **257**, 11808–11815.